# Dopamine-2 receptor extracellular N-terminus regulates receptor surface availability and is the target of human pathogenic antibodies from children with movement and psychiatric disorders

**DOI:** 10.1186/s40478-016-0397-1

**Published:** 2016-12-01

**Authors:** Nese Sinmaz, Fiona Tea, Deepti Pilli, Alicia Zou, Mazen Amatoury, Tina Nguyen, Vera Merheb, Sudarshini Ramanathan, Sandra T. Cooper, Russell C. Dale, Fabienne Brilot

**Affiliations:** 1 Brain Autoimmunity group, Institute for Neuroscience and Muscle Research, The Kids Research Institute at the Children’s Hospital at Westmead, University of Sydney, Westmead, NSW 2145 Australia; 2 Membrane Repair group, Institute for Neuroscience and Muscle Research, The Kids Research Institute at the Children’s Hospital at Westmead, University of Sydney, Westmead, NSW 2145 Australia

**Keywords:** Dopamine-2 receptor, N-terminus, Epitope, Autoantibody, Autoimmune movement and psychiatric disorders, Pathogenicity

## Abstract

Anti-Dopamine-2 receptor (D2R) antibodies have been recently identified in a subgroup of children with autoimmune movement and psychiatric disorders, however the epitope(s) and mechanism of pathogenicity remain unknown. Here we report a major biological role for D2R extracellular N-terminus as a regulator of receptor surface availability, and as a major epitope targeted and impaired in brain autoimmunity. In transfected human cells, purified anti-D2R antibody from patients specifically and significantly reduced human D2R surface levels. Next, human D2R mutants modified in their extracellular domains were subcloned, and we analyzed the region bound by 35 anti-D2R antibody-positive patient sera using quantitative flow cytometry on live transfected cells. We found that N-glycosylation at amino acids N5 and/or N17 was critical for high surface expression in interaction with the last 15 residues of extracellular D2R N-terminus. No anti-D2R antibody-positive patient sera bound to the three extracellular loops, but all patient sera (35/35) targeted the extracellular N-terminus. Overall, patient antibody binding was dependent on two main regions encompassing amino acids 20 to 29, and 23 to 37. Residues 20 to 29 contributed to the majority of binding (77%, 27/35), among which 26% (7/27) sera bound to amino acids R20, P21, and F22, 37% (10/27) patients were dependent on residues at positions 26 and 29, that are different between humans and mice, and 30% (8/27) sera required R20, P21, F22, N23, D26, and A29. Seven patient sera bound to the region 23 to 37 independently of D26 and A29, but most sera exhibited N-glycosylation-independent epitope recognition at N23. Interestingly, no evident segregation of binding pattern according to patient clinical phenotype was observed. D2R N-terminus is a central epitope in autoimmune movement and psychiatric disorders and this knowledge could help the design of novel specific immune therapies tailored to improve patient outcome.

**Electronic supplementary material:**

The online version of this article (doi:10.1186/s40478-016-0397-1) contains supplementary material, which is available to authorized users.

## Introduction

Dopamine receptor expression and innervation are prominent in the brain, and are involved in the regulation of neuromuscular and psychological functioning, including gross and fine motor control, behaviour, learning, and working memory [3]. In humans, dopamine-2 receptor (D2R) is one of five dopamine receptors (DR), which are rhodopsin-like seven transmembrane G-protein coupled receptors (GPCR) belonging to the catecholamine subfamily. The five subtypes of DR are divided into D1-like group (D1R, D5R), and D2-like group (D2R, D3R, D4R) based on their structural, biochemical, and pharmacological properties [2, 3]. Dopamine receptors share similar structural features, including seven transmembrane domains, an extracellular N-terminal domain, three extracellular loops, three intracellular loops, and an intracellular C-terminal domain [2, 37]. D1- and D2-like receptors have high expression in the cortex, hippocampus, basal ganglia, such as the striatum and the substantia nigra, and they differ in their ability to modulate cyclic AMP (cAMP) production [3].

Dopaminergic dysregulation has been related to multiple disorders, such as schizophrenia, bipolar disorders, depression, and movement disorders such as Parkinsonism and Tourette syndrome [3, 19]. The pathophysiology of some of these disorders is poorly understood, however the immune system may play a role in some patients with these conditions. Recently, a subgroup of pediatric patients was found to harbor specific autoantibodies against D2R, and that association was accompanied by a spectrum of movement and psychiatric disorders of suspected autoimmune aetiology [13]. They are detected in the majority of children with basal ganglia encephalitis (an inflammatory basal ganglia syndrome with dystonia-parkinsonism), a significant minority of Sydenham chorea (a post-streptococcal autoimmune syndrome), and a small subgroup of patients with Tourette syndrome and acute onset psychosis. Common to all patients is the presence of movement and psychiatric disturbances. These patients with autoimmune movement and psychiatric disorders often have post-infectious onset and improve with early immune suppressive or immune modulating therapies [13, 41], suggesting that an immune-mediated process occurs.

Although the physiology and function of the DRs are relatively well known, little is understood of D2R extracellular N-terminal domain and its role in disease. In the present study, we explored the function and influence of D2R N-terminal residues and N-glycosylation sites responsible for protein export to the cell surface. Furthermore, we define specific residues of the D2R N-terminus as targets of pathogenic autoantibodies in movement and psychiatric disorders [48], providing novel therapeutic targets for patients affected by anti-D2R antibody-associated autoimmune disorders.

## Materials and methods

### Patient and controls samples

Serum was collected from pediatric anti-D2R antibody-positive patients (*n* = 35) with autoimmune movement and psychiatric disorders (MPD) who have been recruited locally, interstate, and internationally: basal ganglia encephalitis (BG, defined as in [13], *n* = 15), post-herpes simplex virus encephalitis autoimmune movement disorder (HEM, defined as in [35], *n* = 3), Sydenham chorea (SC defined as [8], *n* = 12), first episode of psychosis (FEP defined as [41], *n* = 2), and Tourette syndrome (TS, fulfilled DSM-IV criteria for Tourette syndrome, *n* = 3) (Table 1). Some patients (*n* = 29) had been identified as anti-D2R antibody-positive in previous studies [13, 35, 41, 44] (Table 1) and additional patients (*n* = 6) were newly identified as anti-D2R antibody-positive using our flow cytometry live cell-based assay [1]. All patients were defined positive as >3SD above the mean of 32 non-inflammatory controls. Ethics approval for this study was granted by the Sydney Children’s Hospital Network Human Research Ethic Committee (HREC 2007/035, HREC 09/CHW/56, and HREC 12/SCHN/395), and written informed consent from all patients was obtained. BG sera used in antibody studies was from the first week of acute admission, and before immune therapy. SC sera were taken during active chorea in all patients. Blood sampling for FEP sera occurred at median of 5 weeks and mean of 14.3 weeks after psychosis symptom onset [41]. TS patients had active tic disorders at the time of serum sampling (Table 1).

**Table 1:** Clinical and demographic characteristics of children with movement and psychiatric disorders

Disease	Patient number	Age median (range)	Reference
Sydenham Chorea	12	9.5 (1–13)	[13]
Basal Ganglia encephalitis	15	6.5 (2–11)	[13]
Post-herpes simplex virus encephalitis autoimmune movement disorder	3	4 (1–6)	[35]
Tourette Syndrome	3	8 (5–8)	[13]
First episode psychosis	2	16 (15–17)	[41]

**Fig. 1:**
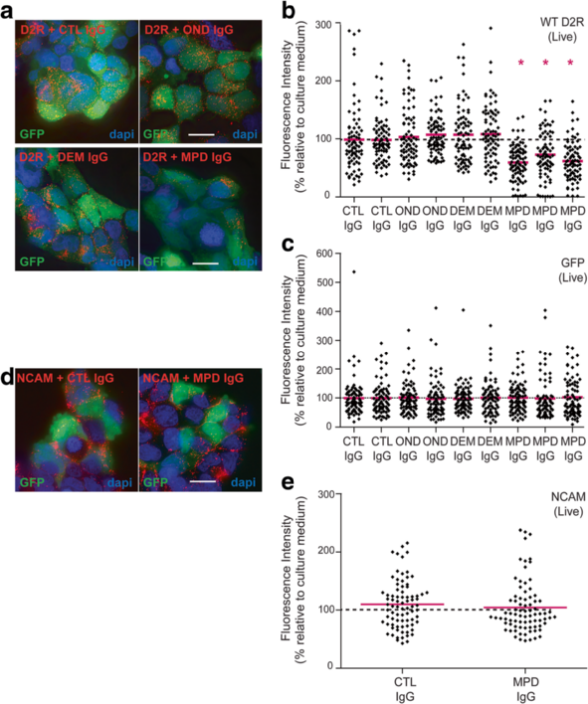
Patient anti-D2R IgG antibodies induce receptor internalization and decrease D2R surface density. **a**, **b** Anti-D2R antibody-positive movement and psychiatric disorder (MPD, *n* = 3) protein G-purified IgG from patients with similar ΔMFIs (14,388; 14,789; and 18,740, Fig. 2e) induces a downregulation of surface D2R on live cells compared to IgG purified from control (CTL, *n* = 2), other neurological disorders (OND, *n* = 2), and demyelinating diseases (DEM, *n* = 2). **c** Anti-D2R antibody-positive MPD (*n* = 3) protein G-purified IgG-incubated on live cells have a similar expression of cytoplasmic GFP compared to IgG purified from CTL (*n* = 2), OND (*n* = 2), and DEM (*n* = 2). **d**, **e** MPD protein G-purified IgG and IgG purified from CTL have no effect on surface NCAM (expressed in all HEK293 cells). Representative images are shown (volume projection of entire Z-stack). Nuclei stained with dapi (scale bar = 25μm). 80 different cells (1 cell = 1 diamond) for each control and patient out of three independent experiments are shown. Results are expressed as fluorescence intensity relative to culture medium (100%) after normalization to GFP and red bars represent mean. Data was analyzed by Mann-Whitney *U* test, *P < 0.0001

**Fig. 2:**
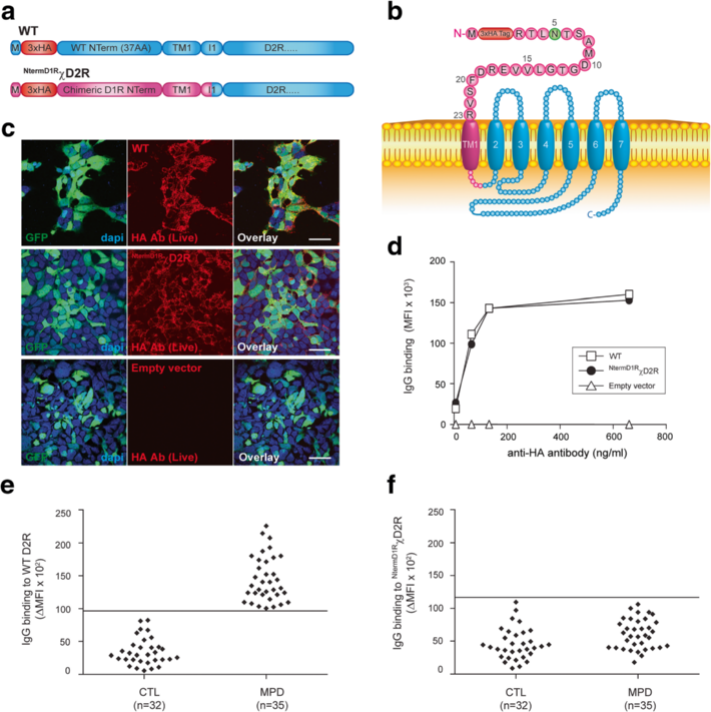
Patient antibodies do not bind to the three extracellular loops of D2R or to D1R extracellular N-terminus. **a**, **b** Open reading construct of WT D2R, and ^NTermD1R^χD2R constructs in which the extracellular N-terminus of D2R was swapped for the extracellular N-terminus of D1R. M = methionine; AA = amino acid; Red = 3x haemagglutinin (HA) tag; Blue = D2R sequence; Pink = D1R sequence; TM1 = first transmembrane domain; I1 = first intracellular loop; Green = N-glycosylation site. **c** Confocal images after live immunolabeling of WT D2R or ^NTermD1R^χD2R-transfected HEK293 cells using an anti-HA antibody showed similar levels of cell surface expression for both constructs (scale bar = 50 μm). **d** Similar cell surface expression of WT D2R and ^NTermD1R^χD2R constructs was also confirmed by flow cytometry on live cells. No D2R expression from empty vector was observed. Representative data out of three independent experiments is shown. **e** Sera from controls (CTL; *n* = 32) and movement and psychiatric disorders (MPD; *n* = 35) were incubated with empty vector- and WT D2R-transfected live HEK293 cells at 1:50 dilution, followed by AF647-conjugated anti-human IgG secondary antibody, and flow cytometry analysis. Anti-D2R antibody was detected in 35/35 MPD and 0/32 controls. **f** Sera from CTL (*n* = 32) and anti-D2R antibody-positive MPD (*n* = 35) were incubated with empty vector- and ^NTermD1R^χD2R-transfected live HEK293 cells at 1:50 dilution, followed by AF647-conjugated anti-human IgG secondary antibody, and flow cytometry analysis. Antibodies against the three D2R extracellular loops or the D1R extracellular N-terminus were detected in 0/35 MPD and 0/32 controls. ∆ mean fluorescence intensity (∆MFI) was calculated using MFI obtained with empty vector- and WT D2R- and ^NTermD1R^χD2R-transfected HEK293 cells. Positivity threshold was determined by ∆MFI of three SD above the mean of CTL (solid line on graph). Representative dot plot out of three experiments is shown

Control sera (*n* = 32) shown in Fig. 1 were obtained from healthy children (CTL), children with other neurological disease, such as epilepsy and cerebral palsy (OND), and first episode of demyelination (DEM). Control (CTL) sera (*n* = 32) shown in Fig. 2 were obtained from children with epilepsy, cerebral palsy, neurometabolic disease and neurodegenerative disorders (median age 11 years, range 2-14).

Patient sera had immunoglobulin G (IgG) concentrations measured by nephelometry (BN ProSpec, Siemens, Germany), and IgG values were within the normal range (6.2-14.4g/l).

### Human dopamine receptor mutants

**Fig. 3:**
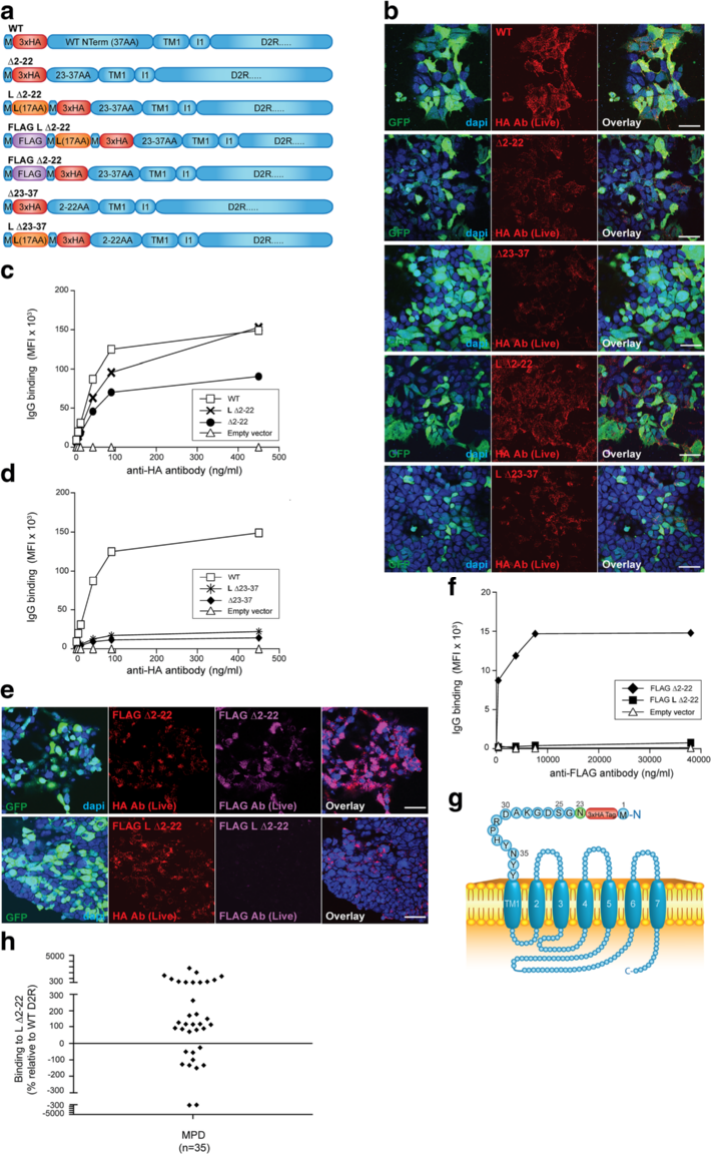
The majority of patient antibodies bind to a region encompassing amino acids 23-37 of D2R extracellular N-terminus. **a** Open reading construct of WT D2R, Δ2-22 (D2R missing amino acids 2-22 of extracellular N-terminus), L Δ2-22 (Δ2-22 with additional cleavable Lucy (L) signal peptide), FLAG L Δ2-22, FLAG Δ2-22, Δ23-37 D2R (D2R missing amino acids 23-37 of D2R extracellular N-terminus), L Δ23-37 D2R (Δ23-37 with additional cleavable L signal peptide). M = methionine; AA = amino acid; Red = 3x haemagglutinin (HA) tag; Blue = D2R sequence; Purple = FLAG sequence; TM1 = first transmembrane domain; I1 = first intracellular loop; Green = N-glycosylation site. **b** Confocal images after live immunolabeling of WT D2R and mutant-transfected HEK293 cells using an anti-HA antibody showed decreased cell surface expression of Δ2-22 and Δ23-37 compared to WT D2R. Introduction of a L signal peptide in Δ2-22, but not in Δ23-37, significantly increased surface expression to levels observed in WT D2R (scale bar = 50 μm). **c** Increase of surface expression on live L Δ2-22-transfected HEK293 cells was confirmed by flow cytometry. Representative data out of three independent experiments is shown. **d** Lack of rescue of surface expression on live L Δ23-37-transfected HEK293 cells was confirmed by flow cytometry. Representative data out of three independent experiments is shown. **e**, **f** Confocal imaging and flow cytometry analyses after live immunolabeling of FLAG L Δ2-22- and FLAG Δ2-22-transfected HEK293 cells showed successful L signal peptide cleavage in FLAG L Δ2-22-transfected HEK293 cells (GFP staining not shown in overlay) (scale bar = 50 μm). **g** Schematic of L Δ2-22 D2R at cell surface. Red = 3xHA tag; Green = N-glycosylation site; Blue = D2R sequence. **h** Sera from anti-D2R antibody-positive movement and psychiatric disorders (MPD, *n* = 35) were incubated with empty vector-, WT D2R-, and L Δ2-22-transfected live HEK293 cells at 1:50 dilution, followed by AF647-conjugated anti-human IgG secondary antibody, and flow cytometry analysis. Percentage of sera binding to L Δ2-22 (MFI %) was calculated using the formula described in Material and Methods. 71% patients (25/35) recognized L Δ2-22 (518 ± 683%, *n* = 25), whereas 29% (10/35) showed no immunoreactivity to amino acids 23-37. Binding threshold is represented by solid line on graph. Representative data out of three experiments is shown

**Fig. 4:**
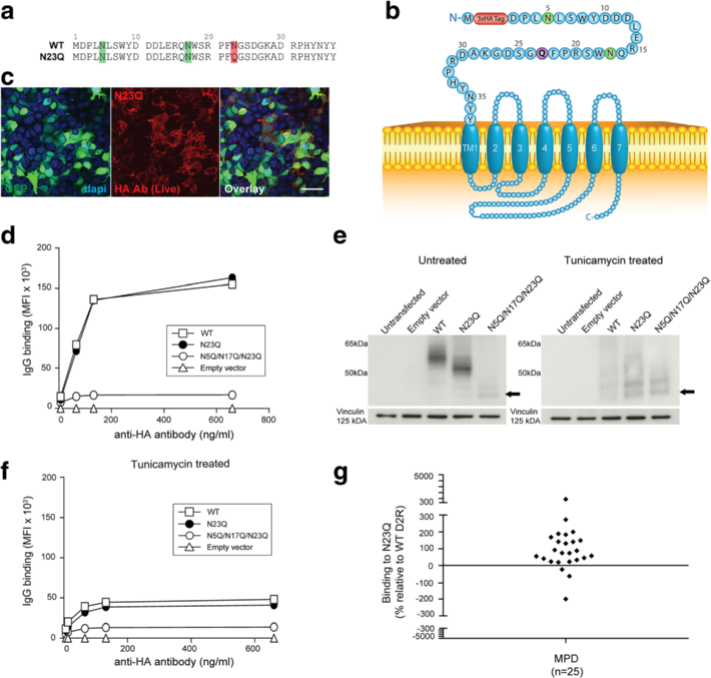
Point mutation of D2R third N-glycosylation site does not abolish anti-D2R antibody binding. **a** Alignment of extracellular N-terminal 37 amino acids of N23Q and WT D2R. Green = putative N-glycosylation sites; Red = N to Q point mutation. **b** Schematic of N23Q at cell surface. Red = 3xHA tag; Green = N-glycosylation site; Blue = D2R sequence; Purple = point mutation. **c**, **d** Confocal images and flow cytometry analysis after live immunolabeling of WT D2R and N23Q-transfected HEK293 cells with an anti-HA antibody showed a similar level of surface expression for both constructs (scale bar = 50 μm). No D2R expression from empty vector was observed. Reduced surface expression of N5Q/N17Q/N23Q was observed. Representative data out of three independent experiments is shown. **e** Loss of N-linked glycan and decrease in molecular weight was detected by western blot analysis in untreated (left panel) and tunicamycin-treated (right panel) WT D2R-, N23Q-, and N5Q/N17Q/N23Q-transfected HEK293 cells. Tunicamycin treatment led to blockade of glycosylation in all mutant-transfected cells. Representative data out of three experiments is shown. **f** Deglycosylation led to impairment in trafficking and decreased cell surface expression of WT D2R, N23Q, and N5Q/N17Q/N23Q. **g** Sera from anti-D2R antibody-positive movement and psychiatric disorders (MPD, *n* = 25) were incubated with empty vector-, WT D2R-, and N23Q-transfected live HEK293 cells at 1:50 dilution, followed by AF647-conjugated anti-human IgG secondary antibody, and flow cytometry analysis. Percentage of sera binding to N23Q (MFI %) was calculated using the formula described in Material and Methods. 88% patients (22/25) recognized N23Q, whereas 12% (3/25) showed no immunoreactivity to N23Q. Binding threshold is represented by solid line on graph. Representative data out of three experiments is shown

**Fig. 5:**
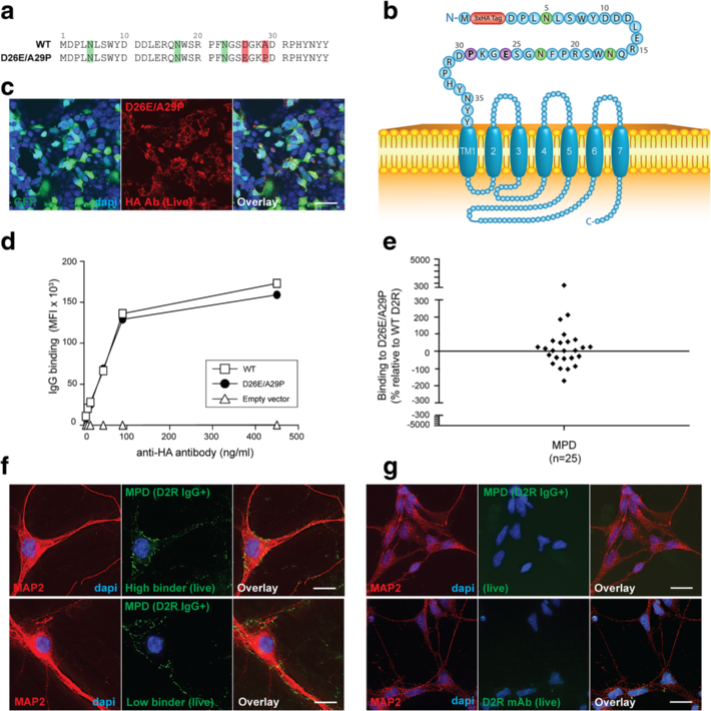
Human antibodies bind to mouse D2R extracellular N-terminus with a lower immunoreactivity. **a** Amino acid sequences of extracellular N-terminus of mouse D2R (D26E/A29P) and human WT D2R. Green = putative N-glycosylation sites; Red = amino acid change between species. **b** Schematic of D26E/A29P at cell surface. Red = 3xHA tag; Green = N-glycosylation site; Blue = D2R sequence; Purple = point mutation. **c** Confocal images after live immunolabeling of D26E/A29P-transfected HEK293 cells using an anti-HA antibody showed high levels of cell surface expression (scale bar = 50 μm). **d** Similar cell surface expression between WT D2R and D26E/A29P constructs was also confirmed by flow cytometry on live cells. No D2R expression from empty vector was observed. Representative data out of three independent experiments is shown. **e** Sera from anti-D2R antibody-positive movement and psychiatric disorders (MPD, *n* = 25) were incubated with empty vector-, WT D2R -, and D26E/A29P-transfected live HEK293 cells at 1:50 dilution, followed by AF647-conjugated anti-human IgG secondary antibody, and flow cytometry analysis. Percentage of sera binding to D26E/A29P (MFI %) was calculated using the formula described in Materials and methods. 60% patients (15/25) recognized D26E/A29P, whereas 40% (10/25) showed no immunoreactivity to D26E/A29P. Binding threshold is represented by solid line on graph. Representative data out of three experiments is shown. **f** Patient sera stained live murine hippocampal neurons, but no difference was observed between high or low serum binders to D26E/A29P mutant. **g** Patient sera did not stain live human neural stem cell-derived neurons, probably because these neurons did not express D2R on cell surface when immunolabeled with commercial anti-D2R antibody (scale bar = 25 μm)

**Fig. 6:**
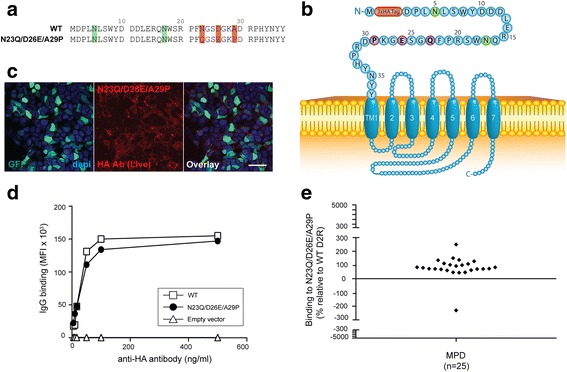
Patient antibody recognise mouse D2R when N23 is deglycosylated. **a** Amino acid sequences of extracellular 37 amino acids of N23Q/D26E/A29P and WT D2R. Green = putative N-glycosylation sites; Red = amino acid changes. **b** Schematic of N23Q/D26E/A29P at cell surface. Red = 3xHA tag; Green = N-glycosylation site; Blue = D2R sequence; Purple = point mutation. **c** Confocal images after live immunolabeling of N23Q/D26E/A29P- transfected HEK293 cells using an anti-HA antibody (scale bar = 50 μm). **d** Similar cell surface expression of N23Q/D26E/A29P and WT D2R constructs was confirmed by flow cytometry on live cells. No D2R expression from empty vector was observed. Representative data out of three independent experiments is shown. **e** Sera from anti-D2R antibody-positive movement and psychiatric disorders (MPD, *n* = 25) were incubated with empty vector-, WT D2R -, and N23Q/D26E/A29P-transfected live HEK293 cells at 1:50 dilution, followed by AF647-conjugated anti-human IgG secondary antibody, and flow cytometry analysis. Percentage of sera binding to N23Q/D26E/A29P (MFI %) was calculated using the formula described in Material and Methods. 96% patients (24/25) recognized N23Q/D26E/A29P, whereas 4% (1/25) showed no immunoreactivity to N23Q/D26E/A29P. Binding threshold is represented by solid line on graph. Representative data out of three experiments is shown

**Fig. 7:**
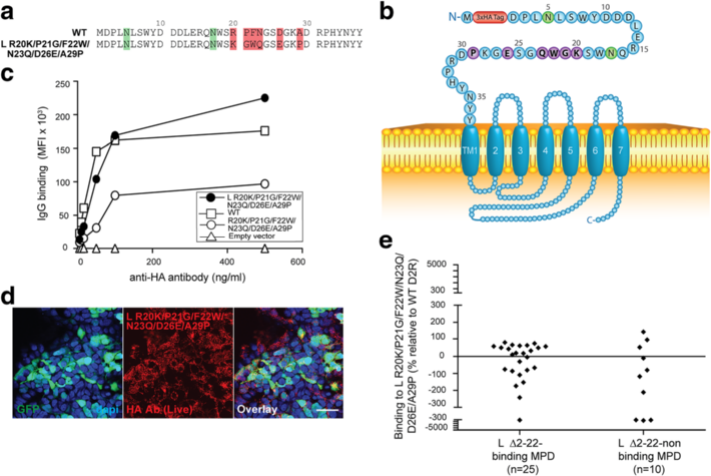
Extracellular N-terminal amino acids 20-22 contribute to a major binding region. **a** Amino acid sequences of extracellular 37 amino acids of R20K/P21G/F22W/N23Q/D26E/A29P and WT D2R. Green = putative N-glycosylation sites; Red = amino acid changes. **b** Schematic of R20K/P21G/F22W/N23Q/D26E/A29P at cell surface. Red = 3xHA tag; Green = N-glycosylation site; Blue = D2R sequence; Purple = point mutation. **c** Similar cell surface expression of L R20K/P21G/F22W/N23Q/D26E/A29P and WT D2R constructs was observed by flow cytometry on live cells, whereas R20K/P21G/F22W/N23Q/D26E/A29P (without L signal peptide) has significantly decreased surface expression. No D2R expression from empty vector was observed. Representative data out of three independent experiments is shown. **d** Confocal images after live immunolabeling of L R20K/P21G/F22W/N23Q/D26E/A29P transfected HEK293 cells using an anti-HA antibody (scale bar = 50 μm). **e** Sera from anti-D2R antibody-positive movement and psychiatric disorders (MPD) that bind L Δ2-22 (*n* = 25) or not (*n* = 10) were incubated with empty vector-, WT D2R-, and L R20K/P21G/F22W/N23Q/D26E/A29P-transfected live HEK293 cells at 1:50 dilution, followed by AF647-conjugated anti-human IgG secondary antibody, and flow cytometry analysis. Percentage of sera binding to L R20K/P21G/F22W/N23Q/D26E/A29P (MFI %) was calculated using the formula described in Material and Methods. Among the group that bound L Δ2-22, 56% patients (14/25) recognized L R20K/P21G/F22W/N23Q/D26E/A29P, whereas 44% (11/25) showed no immunoreactivity to the mutant. Among the group that did not bind L Δ2-22, 7/10 (70%) did not recognize L R20K/P21G/F22W/N23Q/D26E/A29P mutant, whereas 3/10 (30%) did bind to it. Binding threshold is represented by solid line on graph. Representative data out of three experiments is shown

Serum binding to human wild-type D2R (WT D2R), and seven D2R mutants (^NTermD1R^χD2R, L Δ2-22, N5Q/N17Q/N23Q, N23Q, D26E/A29P, N23Q/D26E/A29P and L R20K/P21G/F22W/N23Q/D26E/A29P) was analyzed. The mutations are shown in Figs. 2, 3, 4, 5, 6, and 7. All patient sera recognized the native conformation of WT D2R expressed on the surface of live cells. To discriminate between binding to the D2R extracellular N-terminus or the extracellular loops, we created the chimera ^NTermD1R^χD2R, in which the entire extracellular N-terminus of D2R was substituted with the one of D1R, so we could analyse antibody immunoreactivity to D2R extracellular loops. Additionally, we created ∆2-22 D2R (deletion of amino acids 2-22 of the N-terminal extracellular domain), and ∆23-37 D2R (deletion of amino acids 23-37 of the N-terminal extracellular domain) to define anti-D2R antibody binding to either the first or second half of D2R N-terminus. However, we found that the level of surface expression for both constructs were low compared to WT D2R. Therefore we subcloned L ∆2-22 D2R and L ∆23-37 D2R with a cleavable signal peptide, called Lucy (L), to aid in cell surface expression. In order to determine the contribution of N-linked glycosylation in antibody recognition, we created N5Q/N17Q/N23Q, in which all glycosylation sites (N5, N17, and N23) were mutated, and N23Q D2R mutant, in which only one glycosylation site at position N23 was mutated. As some human autoantibodies do not recognize mouse equivalent antigen, for example human anti-myelin oligodendrocyte glycoprotein (MOG) antibodies do not bind to mouse MOG [33], we generated the mutant D26E/A29P to murinize D2R extracellular N-terminus. Based on results observed after binding to D26E/A29P and N23Q, the triple mutant N23Q/D26E/A29P was generated. As there is a high level of conservation of amino acids 20 to 23 in mammals (Additional file 1: Figure S3), we also generated L R20K/P21G/F22W/N23Q/D26E/A29P mutant with six point mutations in the extracellular N-terminus.

### Molecular cloning of human D2R mutants

Extracellular N-terminus haemagglutinin (HA) tagged human D1R and D2R were obtained from the Missouri S&T cDNA Resource centre (www.cdna.org). We used HA-tagged Syntaxin 4 as a control to exclude immunoreactivity of sera against the HA tag. All sera used in this study were reactive to human D2R, but not to the HA tag ([13], data not shown). Furthermore, studies using HA-tagged GPCRs have shown that the presence of the HA tag at the start of the extracellular N-terminus did not interfere with receptor ligand binding, and receptor activation and signaling [42, 43]. Human D1R and D2R cDNA were subcloned into pIRES2-GFP vector (Clontech), a bicistronic plasmid containing an internal ribosome entry site (IRES), allowing simultaneous expression of green fluorescence protein (GFP) and the antigen of interest, as described previously [1]. To obtain ^NTermD1R^χD2R mutant, we subcloned the extracellular N-terminus of D1R from pIRES2-GFP HA D1R construct into pIRES2-GFP HA D2R. Synthesized mini D2R cDNA (Blue Heron) were subcloned into pIRES2-GFP HA D2R using NheI and PstI to generate pIRES2-GFP HA Δ2-22 D2R, pIRES2-GFP HA Δ23-37 D2R, pIRES2-GFP HA N23Q D2R, pIRES2-GFP HA N5Q/N17Q/N23Q D2R, pIRES2-GFP HA D26E/A29P D2R, pIRES2-GFP HA N23Q/D26E/A29P D2R, and pIRES2-GFP HA R20K/P21G/F22W/N23Q/D26E/A29P D2R. The cleavable signal peptide L (ATGAGACCCCAGATCCTGCTGCTCCTGGCCCTGCTGACCCTAGGCCTGGCT) [47] was inserted by PCR in pIRES2-GFP HA Δ2-22 D2R, pIRES2-GFP HA Δ23-37 D2R, and pIRES2-GFP HA R20K/P21G/F22W/N23Q/D26E/A29P D2R constructs using two oligonucleotides containing the L signal peptide 5’-AGATCCGCTAGCGTTTAAACTTAAGCTTGGTACCACCATGAGACCCCAGATCCTGCTGCTCCTGGCCCTGCTGACCCTAGGCCTGGCTATGTACCCATACGATGTTCCAGA-3’ and 5’-GGTAGTTGGTGGTGGTCTGCAGCGCC-3’. The oligonucleotides used to create pIRES2-GFP HA FLAG Δ2-22 D2R were 5’-AGATCCGCTAGCGTTTAAACTTAAGCTTGGTACCACCATGGACTACAAGGACGACGATGACAAGATGTACCCATACGATGTTCCAGAT-3’; 5’-GGTAGTTGGTGGTGGTCTGCAGCGCC-3’ and 5’-AGATCCGCTAGCGTTTAAACTTAAGCTTGGTACCACCATGGAC-TACAAGGACGACGATGACAAGATGAGACCCCAGATCCTGCTGCT-3’; 5’-GGTAGTTGGTGGTGGTCTGCAGCGCC-3’ for pIRES2-GFP HA FLAG L Δ2-22 D2R constructs, which included FLAG tag (ATGGACTACAAGGACGACGATGACAAG). Purified plasmids were sequenced at the Australian Genome Research Facility to confirm correct plasmid sequences.

### Analysis of antibody binding using flow cytometry live cell-based assay

Human D2R constructs were transiently transfected and expressed in human embryonic kidney 293 (HEK293) cells as previously described [13, 41]. All mutant transfections led to a transfection yield of 30-40%. Surface expression of D2R constructs was confirmed by flow cytometry on live cells. In all experiments, expression of WT D2R reached 156,000 ± 29,486 mean fluorescence intensity (MFI) at 100 ng/ml of anti-HA antibody and 162,429 ± 8174 MFI at 500 ng/ml. If D2R mutant surface expression was below 80% of the WT D2R, then a L signal peptide was attached. If the addition of the L signal peptide did not successfully promote D2R mutant expression above 80% of WT D2R, they were not included in our analysis (i.e. L Δ23-37 mutant). A flow cytometry live cell-based assay was used to detect antibody binding from patient sera to transfected HEK293 cells. In Fig. 2e, experiments were performed as previously described [1, 5, 6, 41]. Additionally, six sera from D2R antibody-positive patients (selected for adequate volume of serum available) were serially incubated with five wells of live WT D2R- and ^NTermD1R^χD2R-transfected HEK293 cells [41]. Immunolabeling was assessed by flow cytometry cell-based assay. To analyze epitope binding, each anti-D2R antibody-positive serum was separately incubated concomitantly with three types of cells in the same experiment: cells transfected with human WT D2R, empty vector, and D2R mutant. Binding percentage was determined using the formula: $binding\kern0.5em \%\kern0.5em =\kern0.5em \frac{MFI\left(D2R\kern0.5em  mutant\right)-MFI\left( empty\kern0.5em  vector\right)}{MFI\left(WT\kern0.5em D2R\right)-MFI\left( empty\kern0.5em  vector\right)}\times 100\%.$ Highly transfected cells with high GFP expression between 10^3^-10^4^ were analyzed. MPD patient sera were always positive for the difference in MFI between WT D2R and empty vector (denominator in formula). Therefore negative binding percentages reflect the inability of anti-D2R antibodies from the patient to bind to the D2R mutant. Binding of patient sera to our different constructs was analyzed in three independent experiments. Surface expression curves and dot plots were generated using Prism software version 6 (GraphPad Software, La Jolla, CA). Flow cytometry data were acquired on a BDLSRII (BDBiosciences) flow cytometer, and analysis was performed using Flowjo v7.5 software (TreeStar, Ashland, Oregon) and Excel (Microsoft, Redmond, Washington).

### Culture and differentiation of human neural stem cell-derived neurons

GIBCO human neural stem cells (H9 hESC-derived) (Life Technologies) were cultured and differentiated into neurons according to manufacturer’s instructions. Briefly, neural stem cells were cultured on CellStart-coated (1:100) culture dish in complete serum-free human neural stem cell culture medium (StemPro NSC SFM). To differentiate neural stem cells into neurons, cells were plated on a poly-L-ornithine and laminin-coated culture dish in complete StemPro NSC SFM. After two days, medium was switched to neural differentiation medium, which was changed 3-4 days later. To expedite differentiation, 0.5 mM of dibutyryl cAMP was added to the differentiation medium daily starting at day 7 of differentiation for 3 days.

### HEK293 cells, primary murine hippocampal, and human neural stem cells immunocytochemistry

HEK293 cells were immunolabeled in live and fixed/permeabilized conditions [13]. Slides were imaged using an inverted confocal microscope (63X 1.4 numerical aperture oil immersion lens) (Leica, Germany) and analyzed using Image J v1.46 software (National Institutes of Health, Bethesda, Maryland). Embryonic day 16.5 mouse hippocampal neurons were cultured as previously described [13, 17]. Immunocytochemistry on live primary murine neurons and human neural stem cell-derived neurons were performed as previously described [13, 41]. Neurons were visualized through 100X 1.4 numerical aperture oil immersion lens with an inverted Olympus IX-70 microscope (DeltaVision Core, Applied Precision, GE Healthcare, Washington) and a CoolSnap QE camera (Photometrics, Tucson, Arizona). Images were acquired as 0.15 μm-thick 40 serial optical sections, then deconvolved using DeltaVision SoftWoRx software, version 5.0.0 (Applied Precision, GE Healthcare), and volume projections of the entire Z-series were generated and overlaid using ImageJ. All procedures on animals were approved by the Kids Research Institute and Children’s Medical Research Institute animal ethics committee and conformed to the published code of practice of the National Health and Medical Research Council of Australia.

### Analysis of surface D2R downregulation

To determine whether human IgG from anti-D2R antibody patients and controls induced downregulation of cell surface D2R, we quantified the fluorescence of surface D2R after incubation of patient or control IgG with live cells. We purified IgG from 50-100 μl of patient sera with similar ΔMFIs (ΔMFIs: 14,388; 14,789; and 18,740 on Fig. 2e) using protein G-agarose and Microcon (Millipore), and obtained from 3.2 to 11.9μg/μl of purified IgG in a total volume of 20μl. We chose WT D2R-transfected HEK293 cells as they are commonly used in neuropharmacology to study the effects of D2R agonists on receptor trafficking and internalization [29]. Surface D2R or Neural Cell Adhesion Molecule (NCAM, surface adhesion molecule used as control) on live WT D2R-transfected HEK293 cells was labelled with anti-HA and anti-NCAM antibodies, respectively, and appropriate secondary antibody for 30 min at room temperature. NCAM- and D2R-labelled live cells were then incubated with 8μg of protein G-purified human IgGs from patient or control sera, or culture medium alone for 30 min or 2 h at room temperature, or 30 min at 37**°**C. After washing, cells were fixed and mounted, and cell surface D2R and NCAM fluorescence was quantified using 3D-deconvolution microscopy. Importantly, both controls and patient-treated cells received the same labelling with anti-HA or anti-NCAM antibody in order to control the impact of antibody binding on the receptor and provide suitable controls. For quantification purposes, we have used 3D-deconvolution algorithm on images acquired through the Deltavision Core microscope, as previously described [14]. Levels of surface D2R and NCAM were calculated by measuring the average intensity per unit volume at the cell surface after all Z-series were summed. D2R values were normalized to GFP values and expressed as mean ± SD of fluorescence intensity relative to the “culture medium alone” condition (100%). For consistency, NCAM was analyzed in GFP-positive HEK293 cells, and values were also normalized to GFP, although all HEK293 cells, transfected or not, expressed surface NCAM. NCAM results were also expressed as fluorescence relative to the “culture medium alone” condition (100%).

### Western blot

D2R mutant-transfected HEK293 cells were lysed at 4°C for 30 min with RIPA buffer (150 mM NaCl, 1% NP-40, 0.5% DOC, 0.1% SDS, 50 mM Tris pH 8) and 5 μl protease inhibitor (PI)/ml. Protein concentration in supernatant was measured using BCA assay (Thermo Scientific) on a plate reader. For experiments blocking N-linked glycosylation, media of transfected HEK293 cells were supplemented with 2 μg/ml of Tunicamycin. 2.5 μg of total protein was loaded onto SDS gel and separated by gel electrophoresis. The membrane was blocked in 4% milk for 30 min, and incubated with anti-HA antibody (1:1000) and mouse anti-vinculin antibody (1:1000) for 1 h at room temperature then washed three times in 2% milk-PBS-0.2% Tween 20. Next, the membrane was incubated in anti-rabbit horseradish peroxidase (HRP)- and anti-mouse HRP-conjugated secondary antibody (1:1000) for 1 h at room temperature, then developed in ECL after washes.

### Statistical analysis

Mann-Whitney *U* test was used to compare CTL and MPD cohorts in Fig. 1. One-way analysis of variance with a Dunn’s multiple comparison test was carried out to analyze differences in binding percentage between D2R mutants. *P* < 0.05 was considered significant.

## Results

### Human antibodies target surface D2R and purified IgG causes surface D2R downregulation

We have previously shown that a subgroup of pediatric patients with movement and psychiatric disorders (MPD) were anti-D2R antibody-positive and that the antibodies reacted towards an extracellular epitope of surface human D2R, but not against surface D1R [13, 41]. To determine whether anti-D2R IgG antibody would alter levels of surface D2R, we used live transfected HEK293 cells that express high levels of human WT D2R. Firstly, we tagged surface D2R using anti-HA antibody on live WT D2R-transfected HEK293 cells, and then incubated the cells with 8 μg of protein G-purified IgG from controls (CTL), other neurological disorders (OND), demyelinating diseases (DEM) (positive for anti-MOG antibody [14]), MPD anti-D2R antibody-positive sera with similar ΔMFIs, or culture medium alone for 30 min or 2 h at room temperature, or 30 min at 37 °C. Live and healthy looking cells were then fixed and images were acquired using 3D-deconvolution microscopy. We observed a statistically significant decrease of surface D2R after incubation of live cells with MPD IgG from all three anti-D2R antibody-positive patients that were tested after 2 h incubation at room temperature (Fig. 1a, b) (*P* < 0.0001, 59.5 ± 30%, 72.6 ± 40%, and 61.6 ± 32.7%), whereas levels of normalized surface D2R were similar in CTL (98.7 ± 57.4% and 98.3 ± 38.6%) and OND (103.3 ± 48.3% and 107.1 ± 34%) IgG-treated cells (Fig. 1a, b). Importantly, this effect focused on receptor downregulation from the surface as cells were not permeabilized before immunolabeling. To assess the specificity of this effect with MPD IgG, we incubated live anti-HA antibody-tagged WT D2R-transfected HEK293 cells with anti-MOG antibody-positive DEM IgG (106.6 ± 49.9% and 108.2 ± 49.9%), and could not observe any decrease in surface D2R (Fig. 1a, b). Levels of intracytoplasmic GFP were similar in all conditions tested (Fig. 2c). This decrease in surface expression was not observed when cells were incubated for 30 min either at room temperature or at 37 °C (Additional file 1: Figure S1). Furthermore, we also controlled whether levels of surface NCAM, a molecule constitutively expressed at the surface of HEK293 cells, were affected by incubation with IgG from CTL and MPD. Incubation of CTL (109.9 ± 41.4%) and anti-D2R antibody-positive MPD IgG (104.1 ± 44.7%) did not induce any changes in levels of surface NCAM (Fig. 1d, e), suggesting that MPD IgG specifically induced D2R downregulation, and that this effect was through binding of antibodies to extracellular domains of WT D2R.

### Antibodies specifically target the extracellular N-terminal domain of D2R

The extracellular domains of human WT D2R consist of the N-terminus of 37 amino acids, and three extracellular loops. We therefore examined which extracellular epitopes were recognised by MPD autoantibodies, using D2R N-terminal deletion constructs and glycosylation mutants. We first subcloned ^NTermD1R^χD2R in which the entire extracellular N-terminus of D2R was substituted with the one of the closely related D1R (Fig. 2a, b). Surface expression of ^NTermD1R^χD2R was found to be similar to WT D2R (Fig. 2c, d). Next, we tested MPD anti-D2R antibody specificity to ^NTermD1R^χD2R by flow cytometry live cell-based assay. We found that immunolabeling of live WT D2R-transfected cells by ^NTermD1R^χD2R-immunoabsorbed sera was higher compared to their immunoabsorption on WT D2R-transfected cells (Additional file 1: Figure S2), and none of 35 MPD anti-D2R antibody-positive sera (0%) (Fig. 2e) bound to ^NTermD1R^χD2R (Fig. 2f), suggesting that MPD antibodies do not bind to the extracellular N-terminus of D1R or the extracellular loops of D2R.

### Amino acids 23 to 37 include a major binding region for anti-D2R IgG antibodies

To define anti-D2R antibody specificity within the 37 amino acids of D2R extracellular N-terminus, we split the N-terminus into two sections. The first construct, called Δ2-22, only included amino acids 23-37 of the N-terminus followed by the remaining human WT D2R sequence (Fig. 3a). The second construct, Δ23-37, only contained amino acids 2-22 of D2R N-terminus followed by WT D2R sequence (Fig. 3a). Both constructs (Δ2-22 and Δ23-37), each lacking a portion of the D2R N-terminal extracellular domain, showed reduced cell surface expression levels compared to WT D2R (Fig. 3b, c, d). As surface expression influences the analysis of autoantibody epitope binding, we attempted to increase Δ2-22 and Δ23-37 deletion mutant surface expression by using a cleavable signal peptide derived from T regulatory cell type 1 membrane protein, Leucine-Rich Repeat Containing 32 (LRRC32), named Lucy (L) because of its leucine-rich repeat regions [47]. The L signal peptide was inserted before the HA tag in L Δ2-22 and L Δ23-37 (Fig. 3a). The L signal peptide significantly increased Δ2-22 trafficking to the surface: from 68% surface expression for Δ2-22 (Fig. 3c, 107,167 ± 15,590 MFI, *n* = 3) to 101% for L Δ2-22 (Fig. 3c, 160,000 ± 12,832 MFI, *n* = 3) compared to 100% for WT D2R (Fig. 3c, 158,667 ± 2624 MFI, *n* = 3). In contrast, the addition of the L signal peptide to Δ23-37 could not rescue surface expression (Fig. 3b, d), suggesting residues 23-37 are fundamentally important for export of D2R through the secretory pathway to the plasma membrane.

We next determined whether the L signal peptide was cleaved before surface expression in order to assess the risk of non-specific binding of autoantibodies to uncleaved L signal peptide. A FLAG tag was added at the extreme N-terminus of both L Δ2-22 and Δ2-22 (Fig. 3a). Both FLAG L Δ2-22- and FLAG Δ2-22-transfected live HEK293 cells expressed the surface mutant receptor as detected after anti-HA antibody immunolabeling, with FLAG L Δ2-22 D2R showing a higher surface expression compared to FLAG Δ2-22 D2R (Fig. 3e), indicating that the HA tag was present on the mature protein. However, when FLAG L Δ2-22-expressing cells were surface labeled using anti-FLAG antibody, the FLAG epitope was no longer present on the cell surface (Fig. 3e, f), suggesting that the L signal peptide was a functional signal peptide cleaved early in the biosynthetic pathway, and therefore not detectable at the cell surface. Interestingly, L Δ2-22 could not be immunolabeled by a commercial anti-D2R antibody that targets the extracellular N-terminus, suggesting that commercial anti-D2R antibody epitope lies within amino acids 1-22 of D2R, and providing further rationale to use the HA tag to ensure correct surface transport of mutants (Additional file 1: Figure S3).

The successful cleavage of the L signal peptide, accompanied by the increased surface expression of L Δ2-22 (Fig. 3g) to levels similar to WT D2R, enabled its use to reliably assess autoantibody binding to residues 23-37. We found 25/35 of anti-D2R antibody-positive MPD patients (71%) bound to L Δ2-22, inferring that these patients recognized an epitope within residues 23-37 (mean binding: 518 ± 683%, *n* = 25) (Fig. 3h), whereas 10/35 (29%) did not bind L Δ2-22 (mean binding: -171 ± 156%, *n* = 10) (Fig. 3h). Several positive sera had a very high binding (>300%, mean binding: 1015 ± 785%, *n* = 11), suggesting that deletion of amino acids 2-22 could improve antibody access to epitopes. The binding specificity was similar in all disorders analyzed, and no clinical phenotype-specific binding could be observed (data not shown). This data suggests that a major epitope(s) for MPD autoantibodies is contained within the extracellular N-terminus of D2R encompassing residues 23-37.

### Role of D2R N-Glycosylation in anti-D2R antibody binding

To further refine the specific antigenic region within residues 23-37 of D2R, recognised by two-thirds of our MPD cohort, we next examined the effect of N-glycosylation as it has been reported to influence D2R trafficking to the cell membrane [11]. Indeed, targeted mutagenesis of all three putative N-glycosylation sites (N5Q/N17Q/N23Q, Additional file 1: Figure S4a) led to low cell surface expression and intracellular sequestration of the mutant (Additional file 1: Figure S4). However, point mutation of the third N-glycosylation site only (N23Q) (Fig. 4a, b) did not affect cell surface expression, with levels of anti-HA binding similar to WT D2R (Fig. 4c, d), thereby enabling its use to assess patient antibody binding. Western blot analysis confirmed the loss of the N-linked carbohydrate on N23Q mutant (Fig. 4e), with a decrease of apparent protein weight from 57kDa for WT D2R to 55kDa for N23Q in similarly transfected HEK293 cells (Fig. 4e) (ExpPASy Compute pI/Mw tool). N5Q/N17Q/N23Q was only faintly observed as it is cytoplasmic and not membrane-bound, and was not extracted with RIPA lysis buffer (Fig. 4d, e, and Additional file 1: Figure S4b). Treatment with the N-glycosylation inhibitor tunicamycin (inhibitor of *N*-acetylglucosamine transferases) resulted in the loss of N-linked carbohydrates on asparagines in WT D2R and N23Q (Fig. 4e), but also an important loss of surface trafficking (Fig. 4f), irrespective of the numbers of putative N-glycosylation sites. These data confirm the surface trafficking impairment of N5Q/N17Q/N23Q [11]. Importantly, our data demonstrate glycosylation at N23 is not required for D2R export to the cell membrane, whereas putative glycosylations at N5 and N17 would be.

We then tested the 25 patient sera previously shown to bind to L Δ2-22, and found that 22/25 (88%) patients recognized N23Q (mean binding: 139 ± 174%, *n* = 22), whereas 3/25 (12%) patients did not bind to N23Q (mean binding: -96 ± 75%, *n* = 3) (Fig. 4g). Similar to L Δ2-22, binding was not associated to any specific clinical phenotype (data not shown). Thus, glycosylation at N23 is not the likely basis for antigenicity within residues 23-37.

### Human anti-D2R antibodies have reduced binding to mouse D2R N-terminus

Human IgG specific to human antigens do not always bind to the equivalent mouse antigen in demyelinating diseases [33], and this can have consequences for pathogenic studies in animal models. Thus, we created the mutant D26E/A29P to emulate the mouse D2R extracellular N-terminal domain (Fig. 5a, b). Importantly, amino acids D26/A29 are located within amino acids 23-37, the region relevant to patient antibody targeting. We found D26E/A29P to be highly expressed at the cell surface (Fig. 5c) which was comparable to WT D2R (Fig. 5d). Binding analysis using the D26E/A29P mutant showed that some patient sera (10/25, 40%) did not recognize D26E/A29P mutant (mean binding: -69 ± 44%, *n* = 10), whereas 15/25 (60%) patients bound to this construct (mean binding: 87 ± 162%, *n* = 15) (Fig. 5e) with significantly reduced binding compared to N23Q (*P* < 0.05). Therefore, these data suggest that human patient autoantibodies may be species-specific, highlighting the importance of amino acids D26/A29 in the formation of the D2R antigenic determinant.

As immunolabeling of live murine neurons is recommended in the current clinical algorithm for diagnosis of autoantibody-associated disorders [22], we compared the binding of anti-D2R antibody between immunolabeled live murine primary neurons and human neural stem cell-derived neurons (Fig. 5f, g). Patient sera stained murine hippocampal neurons, but no obvious difference was observed between patient sera that had high or low binding to D26E/A29P mutant via the flow cytometry live cell-based assay (Fig. 5f), suggesting that other epitopes could be present in mouse D2R, especially within the extracellular loops. Patient sera did not stain live human neural stem cell-derived neurons (Fig. 5g) that did not express D2R on cell surface (Fig. 5g), supporting the specificity of patient autoantibody binding to D2R.

As N23 is relatively close to D26/A29 in D2R linear sequence, and the expression of D26E/A29P reduced autoantibody binding, we created N23Q/D26E/A29P mutant (Fig. 6a, b), in which the N-glycosylation site at N23 and human amino acids at positions 26 and 29 were mutated. We found N23Q/D26E/A29P was highly expressed at the cell surface (Fig. 6c, d). After analysis of antibody binding, 24/25 (96%) patient sera bound to this D2R mutant (mean binding: 93 ± 43%, *n* = 24) similarly to N23Q (*P* > 0.05, Fig. 4g), and 1/25 (4%) did not recognize this mutant (binding: -230%, *n* = 1) (Fig. 6e).

### Amino acids 20-22 contribute to anti-D2R antibody binding

10/35 of our MPD patient sera immunoreactive to the D2R N-terminal extracellular domain did not recognise L Δ2-22 (Fig. 3h), and likely recognize antigenic determinants within residues 2-22. However, the influence of amino acids 2-22 in antibody binding is difficult to determine due to the lack of surface expression of L Δ23-37 (including amino acids 2-22). Therefore, due to the importance of amino acids 2-22 in receptor trafficking, the need to keep the N-linked glycosylation conservative site (N-X-S/T) of N17, and phylogenetic alignment of D2R in higher vertebrate revealing that amino acids 20-22 are highly conserved within the animal kingdom (Additional file 1: Figure S5), we constructed R20K/P21G/F22W/N23Q/D26E/A29P mutant (Fig. 7a, b), and used amino acid changes found in different species that were unlikely to modify the overall structure of D2R (Additional file 1: Figure S5). Mutations of these six amino acids strongly decreased surface expression compared to WT D2R (Fig. 7c), and L signal peptide was used to double the surface availability of R20K/P21G/F22W/N23Q/D26E/A29P mutant: from 61% (96,000 MFI) to 141% (223,000 ± 4320 MFI) in L R20K/P21G/F22W/N23Q/D26E/A29P compared to WT D2R (100%) (Fig. 7c, d). We then analyzed patient sera previously shown to bind to L Δ2-22 D2R (Fig. 3h), and found that almost half of the patients (11/25, 44%) did not bind to L R20K/P21G/F22W/N23Q/D26E/A29P (mean binding: -137 ± 127%, *n* = 11), whereas 14/25 (56%) recognized the mutant (mean binding: 50 ± 21%, *n* = 14) (Fig. 7e). We tested the 10 patients who did not recognise L Δ2-22 D2R mutant (Fig. 3h) with L R20K/P21G/F22W/N23Q/D26E/A29P D2R as this subgroup of MPD patients could potentially bind to amino acids at positions 20, 21, 22. We found that 7/10 (70%) did not recognize this mutant (mean binding: -266 ± 208%, *n* = 7), whereas 3/10 (30%) did bind to this mutant (mean binding: 98 ± 36%, *n* = 3) (Fig. 7e). These results suggest that antibody binding is strongly dependent on amino acids 20-22.

### Amino acids 20 to 29 form the major region bound by anti-D2R antibodies

**Fig. 8:**
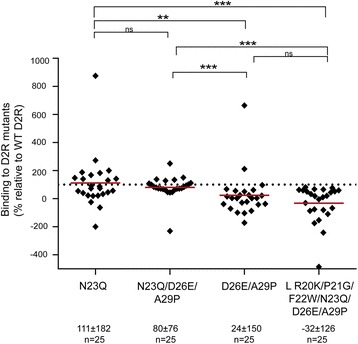
Comparison of antibody binding to four full-length D2R mutants. Binding was similar between N23Q and N23Q/D26E/A29P mutants with WT D2R (100% binding shown as dotted line). Murinization of amino acids 26 and 29 (D26E/A29P) strongly reduced binding. Interestingly, L R20K/P21G/F22W/N23Q/D26E/A29P mutant had the greatest impact on antibody binding. Percentage of sera binding to D2R mutants (MFI %) was calculated using the formula described in Material and Methods. Results are expressed as mean ± SD %. Data was analyzed by one-way ANOVA analysis of variants with a Dunn’s multiple comparison test, *P < 0.05, **P < 0.01, ***P < 0.001

To summarize our data at the cohort level, we compared binding percentages of the four D2R mutants that retained the 37 amino acids of the extracellular N-terminus but included point mutations within this region (Fig. 8). Binding was similar between N23Q (mean binding: 111 ± 182%, *n* = 25, *P* > 0.05) and WT D2R (100% binding). Murinization of amino acids 26 and 29 strongly reduced binding (mean binding: 24 ± 150%, *n* = 25, *P* < 0.01). Interestingly, the deglycosylation was able to compensate the murinization of position 26 and 29, as the N23Q/D26E/A29P mutant (mean binding: 80 ± 76%, *n* = 25, *P* > 0.05) was very similar to N23Q. L R20K/P21G/F22W/N23Q/D26E/A29P mutant had the greatest impact on antibody binding (mean binding: -32 ± 126%, *n* = 25, *P* < 0.001).

**Fig. 9:**
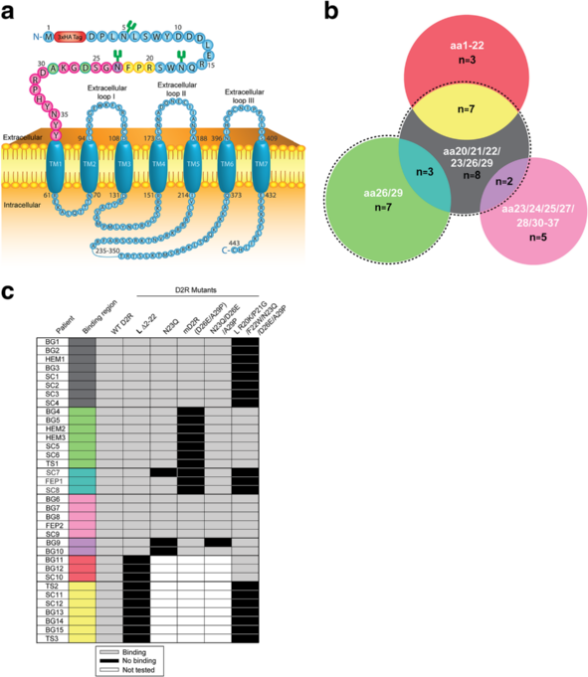
Major binding regions of human anti-D2R antibody on D2R extracellular N-terminus. **a** Schematic of human D2R and extracellular N-terminal amino acids of D2R involved in antibody binding. MPD patient antibody binding was dependent on binding regions centered on residues at positions 20 to 22 (yellow), 26 and 29 (green), and 23 to 37 (magenta). **b** Venn diagram of the main binding regions of 35 pediatric patient sera on human D2R extracellular N-terminus. The black dotted line shown represents binding to residues 20 to 29 by the majority of sera (77%, 27/35). Binding to R20, P21, and F22 is shown in yellow (26%, 7/27), and cyan and green groups represent immunoreactivity to D26 and A29 (37%, 10/27). Seven sera bound to residues 23 to 37 (binding to L Δ2-22 D2R) excluding D26 and A29 (magenta and purple), among which two were dependent on N-glycosylation at N23 (purple). Indeed, the magenta group bound to all D2R mutants modified at R20, P21, F22, N23, D26, and A29, suggesting binding outside these residues. Red shows binding to residues 1 to 19 (9%, 3/35). The Venn diagram was compiled with data summarizing three independent experiments illustrated by black and gray boxes shown in panel c. Amino acids (aa) bound by patient sera are shown in white text. **c** Table of binding patterns of 35 individual anti-D2R antibody-positive patient sera according to D2R mutants (columns) and clinical disorders (rows). Light gray boxes represent binding to mutant and black boxes represent no binding. 10/35 patient sera did not bind to residues 23-37 (L Δ2-22 D2R), and therefore were not tested on mutants encompassing these amino acids (white boxes). No clinical phenotype could be associated with a specific binding pattern. SC, Sydenham chorea; BG, basal ganglia encephalitis; HEM, Post-herpes simplex virus encephalitis autoimmune movement disorder; TS, Tourette syndrome; FEP, first episode psychosis. Colored boxes relate to Venn diagram in panel **b**

Overall, patient antibody binding was dependent on two main regions encompassing amino acids 20 to 29, and 23 to 37 (Fig. 9a, b). The majority of sera (77%, 27/35, black dotted line) bound to a region including amino acids 20 to 29, among which 26% (7/27, yellow) bound to amino acids R20, P21, and F22, 37% (10/27, cyan and green) did not bind to the murinized D2R N-terminus (D26E/A29P mutant), and 30% (8/27, gray) required R20, P21, F22, N23, D26, and A29 (Fig. 9b, c). Seven sera bound to the region 23 to 37 independently of D26 and A29 (magenta and purple), of which two were dependent on N-glycosylation at N23 (purple). Only a minority of sera (9%, 3/35, red) recognized residues 1 to 19 (Fig. 9b, c). Interestingly, we could not observe any evident segregation of binding pattern according to patient clinical phenotype (Fig. 9c).

## Discussion

Using the human glycosylated native conformational D2R, we show the importance of D2R extracellular N-terminus in regulating receptor availability at the cell surface, human anti-D2R antibody binding, and potential pathogenic mechanisms of these antibodies.

Our study highlighted fundamental knowledge about the D2R N-terminus, essential for correct receptor integration into the plasma membrane. Receptor surface trafficking was strongly decreased when N-terminal glycosylation sites were removed. Blocking of N-glycosylation sites via treatment of tunicamycin or obliteration of the three N-glycosylation sites at positions N5, N17, and N23 inhibited receptor transport to the cell surface. These findings support previous studies [11, 28, 34]. However, our study shows that not only are the three glycosylation sites not equal in ensuring correct receptor trafficking, but that their influence is dependent on the amino acids included in the N-terminus. Indeed, single point mutation that only abolished the third N-glycosylation site at residue N23 did not affect surface expression, and the presence of the first two putative N-glycosylated asparagines, N5 and/or N17, could not compensate for the loss of residues 23 to 37. On the other hand, the presence of amino acids 23 to 37 ensured cell surface expression, but only included one glycosylation site (N23). Therefore, the identity, rather than the number of residues, might also play a critical role in surface receptor availability. Furthermore, trafficking to the surface is influenced by the presence of a signal peptide or signal anchor peptide (membrane insertion mediator) in secretory or transmembrane proteins, respectively. Cleavable signal peptides are generally not present in monoamine GPCRs, such as D2R [53], but an uncleaved signal anchor peptide is usually contained within the first transmembrane domain. In the case of human D2R, SignalP-HMM V2.0 predicts the anchor peptide location between Y36 and R61 [36]. Interestingly, removal of the first two putative residues of the predicted 25 residues anchor peptide in Δ23-37 D2R mutant influenced export to the surface that was not boosted by the addition of the Lucy signal peptide.

We assessed 35 anti-D2R IgG antibody-positive patient sera to evaluate the importance of amino acids in D2R N-terminus immunoreactivity. Binding patterns of the majority of patient sera was not associated with any clinical phenotype, was highly dependent on amino acids included between positions 20 to 29 (R20, P21, F22, N23, D26, and A29), and the combination of modifications of residues at these positions had an additive effect that strongly decreased autoantibody binding. While that region was clearly essential for autoantibody binding, only a third of patient sera bound to all of these six amino acids, whereas a quarter of sera preferred R20, P21, F22, and 37% preferred D26 and A29, suggesting that not all of these six amino acids contribute equally to epitope formation. Only a few patients had a strict preference for binding at glycosylated asparagine (N23). This is also the case for other neuroimmune autoantibodies such as anti-AMPAR, -Caspr2, and -MOG antibodies, with only anti-NMDAR and -contactin antibodies displaying a strong binding for N-linked carbohydrate [20, 21, 31, 33, 39, 49].

Differences in binding were observed between human and mouse D2R N-terminal domains. We found that some patients either did not recognize mouse D2R N-terminus, or bound with a lower immunoreactivity, suggesting human autoantibodies may be species-dependent, as previously reported for human anti-MOG antibodies [33, 49]. Furthermore, our data from live mouse primary neurons showed that there was no obvious difference between sera with high and low mouse D2R N-terminus binding, suggesting additional epitopes within the extracellular loops of mouse D2R, or any other posttranslational modifications on neurons, but not present or recognized in human D2R. Although recent guidelines for the identification of antibody-associated brain disorders recommend confirmatory assays on live neurons and rodent tissues [22], our findings also suggest that some human autoantibody-positive patients might be missed when screening with rodent neurons and tissues. The use of primary cultures of human stem cell-derived neurons is also difficult and not the most appropriate replacement for human non neuronal cell lines to investigate binding specificity between species, but also for pathogenic studies, mainly due to their lack of expression of D2R.

Taking the species specificity into account, we investigated the mechanisms of anti-D2R antibody pathogenicity using HEK293 cells expressing conformational human D2R, and an experimental system widely used in neuropharmacology in which cells produced high levels of D2R. We found significant antibody-mediated specific downregulation of surface D2R with patient samples compared to controls. This receptor downregulation may be pathologically relevant in patients with movement and psychiatric disorders. Dopamine binding to D2R is followed by surface D2R downregulation from the cellular membrane leading to dynamic regulation and resensitization [3]. We hypothesize that anti-D2R antibody-mediated downregulation of surface D2R may alter dopamine signalling in patients. Indeed, a similar receptor internalization effect has previously been reported in anti-NMDAR encephalitis and anti-AMPAR encephalitis [15, 24, 32], and is likely to be an important mechanism of pathogenic neuroimmune autoantibodies in general. To support anti-D2R antibody pathogenicity, patients show improvements after treatment with immunotherapies [13]. Animal transfer experiments could validate human anti-D2R antibody pathogenicity, as demonstrated for anti-AQP4 antibody in neuromyelitis optica [30] and anti-NMDAR antibody in encephalitis [45, 54]. However, the difference in epitope binding between human and mouse D2R could influence the relevance of results obtained in animal studies.

Antibody polyclonality, defined by binding to a range of epitopes, is theoretically plausible in human serum samples, and indeed has been suggested in the cases of anti-AQP4 and -MOG antibody-positive patient sera [25, 33]. Although this hypothesis is difficult to demonstrate, the immunoreactivity of the eight patients who bound the six residues 20, 21, 22, 23, 26, and 29 (gray group) could indeed reflect two binding contributions; residues 20 to 23, and residues 26 and 29. Therefore, some binding patterns and the fact that antibody binding could not be completely abrogated may suggest the existence of polyclonality for at least a subgroup of anti-D2R antibody-positive patients.

Further insights into autoantibody epitope recognition have been informed by the resolution of the structural biology of the autoantigen. In particular, three-dimensional models have shown or refuted close conformational proximity of extracellular residues important for autoantibody binding, such as in MOG and AQP4 [33, 40, 51]. Although three-dimensional structures of human D2R [26] and D3R [10] have been reported, neither of these two studies included the N-terminus domain due to the difficulty in interpreting the N-terminus density. Consequently, although epitope conformation is essential for anti-D2R antibody binding as observed by the loss of antibody recognition in peptide-based ELISA (K. Pathmanandavel and F. Brilot, unpublished), spatial contribution of the six main amino acids cannot be deciphered. Interestingly, as in AQP4 [55], formation of supramolecular quaternary high-order receptor mosaics have been predicted for GPCRs, such as D2R [9], and may also influence the specific target of D2R by autoantibodies.

Many mechanisms are likely to cause brain autoimmunity. Molecular mimicry, whereby pathogens express antigens that share structural homology with self-antigens and initiate an immune response against both the pathogen and self-antigen [18, 38], has been reported in other neurological diseases [27, 46, 52]. Furthermore, three recombinant antibodies isolated from a patient with Sydenham chorea displayed cross-reactivity between group A streptococcus and amino acids 1 to 20, and to a lesser extent 21 to 37, of D2R using linear peptides and ELISA [7, 12]. Interestingly, residues 20 to 29, encompassing the six immunodominant amino acids at position 20, 21, 22, 23, 26, and 29, showed sequence homology with residues 55 to 64 of a unknown protein of Penicillium roqueforti FM164, a fungus typically found in soil, decaying organic matter, and industrial production of blue cheese and flavoring agents (Additional file 1: Figure S6). Future investigations are needed to assess the role of molecular mimicry in anti-D2R antibody autoimmunity.

## Conclusion

Defining the pathogenic anti-D2R antibody antigenic region could assist the design of novel specific immunotherapies aimed at preventing interaction between D2R N-terminus and autoantibodies. For example, elimination of autoantigen-specific B cells, recently demonstrated in an animal model of diabetes, has been shown as a potential antigen-specific therapy [23]. Other examples include the blockade of pathogenic antibodies by binding competition with nonpathogenic antibodies [50], and the “decoy antigen therapy”, based on a soluble specific antigen binding to autoantibodies, thus preventing binding to their cognate target [4, 16].

Our study reports for the first time the role of D2R N-terminus in steady state and in disease via its specific binding to pathogenic autoantibodies, and this may be translated into novel treatment options and improved patient outcome.

## Additional file

Additional file 1: Figure S1.
**a**, **b** Anti-D2R antibody-positive movement and psychiatric disorder (MPD) protein G-purified IgG from patient does not induce a downregulation of surface D2R on live cells compared to IgG purified from control (CTL) after 30 min at room temperature or 37 °C. **c** Anti-D2R antibody-positive MPD protein G-purified IgG-incubated on live cells have a similar expression of cytoplasmic GFP compared to IgG purified from control (CTL) after 30 min at room temperature or 37 °C. Representative images are shown (scale bar = 25μm). 15-18 different cells for each control and patient out of two independent experiments are shown. **Figure S2.** Compared to WT D2R-immunoabsorbed MPD sera, ^NTermD1R^χD2R-immunoabsorbed MPD sera had a higher binding to WT D2R-transfected HEK293 cells. Representative data out of two independent experiments is shown. **Figure S3.** Live L Δ2-22 D2R-transfected HEK293 cells were not immunolabeled, by the commercial antibody (Sigma; clone 1B11). Representative data out of three independent experiments is shown. **Figure S4.** **a** Alignment of extracellular N-terminal 37 amino acids of N5Q/N17Q/N23Q and WT D2R. **b** Confocal images of live and fixed/permeabilized N5Q/N17Q/N23Q-transfected HEK293 cells after immunolabeling with anti-HA antibody. Mutations of all three N-glycosylation sites led to low surface expression and intracellular sequestration of the mutant (scale bar = 50 μm). **Figure S5.** Amino acids 1-37 of D2R extracellular N-terminus aligned in 12 different animal species to highlight conserved sequences in mammals (yellow). Amino acid sequences derived from Uniprot database and Uniprot code for the different species. **Figure S6.** Sequence homology between human D2R (20-29) and an unknown protein of the fungus Penicillium roqueforti FM164 (55-64) was identified using the protein–protein Basic Local Alignment Search Tool from National Center for Biotechnology Information. Bolded residues represent identical residues between the two sequences. Underlined residues represent immunodominant amino acids in D2R N-terminus. (PDF 53400 kb)
