# Supplementary material for: Dopamine-2 receptor extracellular N-terminus regulates receptor surface availability and is the target of human pathogenic antibodies from children with movement and psychiatric disorders
Source: Acta Neuropathol Commun. 2016 Dec 1;4:126. doi: 10.1186/s40478-016-0397-1 (PMC5134269; doi:10.1186/s40478-016-0397-1)
Supplement: Additional file 1: Figure S1. — a, b Anti-D2R antibody-positive movement and psychiatric disorder (MPD) protein G-purified IgG from patient does not induce a downregulation of surface D2R on live cells compared to IgG purified from control (CTL) after 30 min at room temperature or 37 °C. c Anti-D2R antibody-positive MPD protein G-purified IgG-incubated on live cells have a similar expression of cytoplasmic GFP compared to IgG purified from control (CTL) after 30 min at room temperature or 37 °C. Representative images are shown (scale bar = 25μm). 15-18 different cells for each control and patient out of two independent experiments are shown. Figure S2. Compared to WT D2R-immunoabsorbed MPD sera, NTermD1RχD2R-immunoabsorbed MPD sera had a higher binding to WT D2R-transfected HEK293 cells. Representative data out of two independent experiments is shown. Figure S3. Live L Δ2-22 D2R-transfected HEK293 cells were not immunolabeled, by the commercial antibody (Sigma; clone 1B11). Representative data out of three independent experiments is shown. Figure S4. a Alignment of extracellular N-terminal 37 amino acids of N5Q/N17Q/N23Q and WT D2R. b Confocal images of live and fixed/permeabilized N5Q/N17Q/N23Q-transfected HEK293 cells after immunolabeling with anti-HA antibody. Mutations of all three N-glycosylation sites led to low surface expression and intracellular sequestration of the mutant (scale bar = 50 μm). Figure S5. Amino acids 1-37 of D2R extracellular N-terminus aligned in 12 different animal species to highlight conserved sequences in mammals (yellow). Amino acid sequences derived from Uniprot database and Uniprot code for the different species. Figure S6. Sequence homology between human D2R (20-29) and an unknown protein of the fungus Penicillium roqueforti FM164 (55-64) was identified using the protein–protein Basic Local Alignment Search Tool from National Center for Biotechnology Information. Bolded residues represent identical residues between the two sequences. Underlined residues represent [file 40478_2016_397_MOESM1_ESM.pdf]

Introduction Figure S1

Patient anti-D2R IgG antibodies did not induce a decrease in D2R surface expression after 30 min at room temperature or 37 °C. Nuclei stained with dapi. Volume projection of entire Z-stack are shown and 1 diamond represents 1 cell. Results are expressed as fluorescence intensity relative to culture medium (100%) after normalization to GFP and red bars represent mean. Data was analyzed by Mann-Whitney U test: not significant.

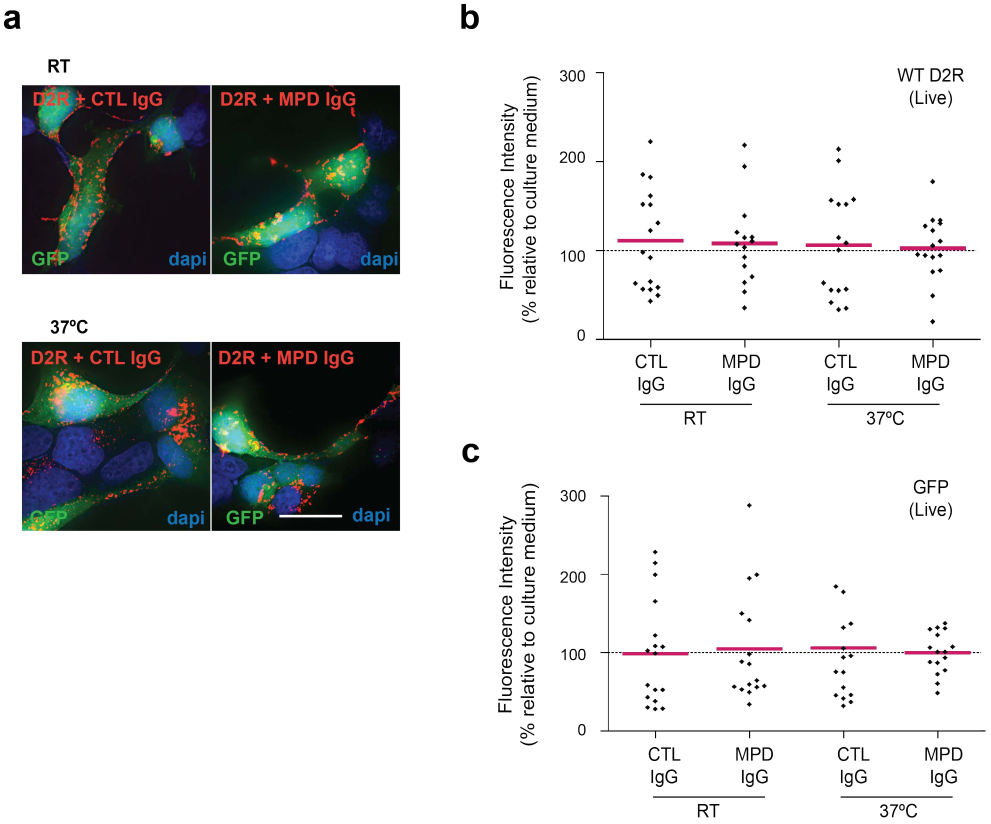

Introduction Figure S2

Immunoreactivity to WT D2R-transfected live HEK293 cells in WT D2R- and $^{NTermD1R}\chi$D2R-immunoabsorbed MPD sera (n = 6) was assessed by flow cytometry live cell-based assay.

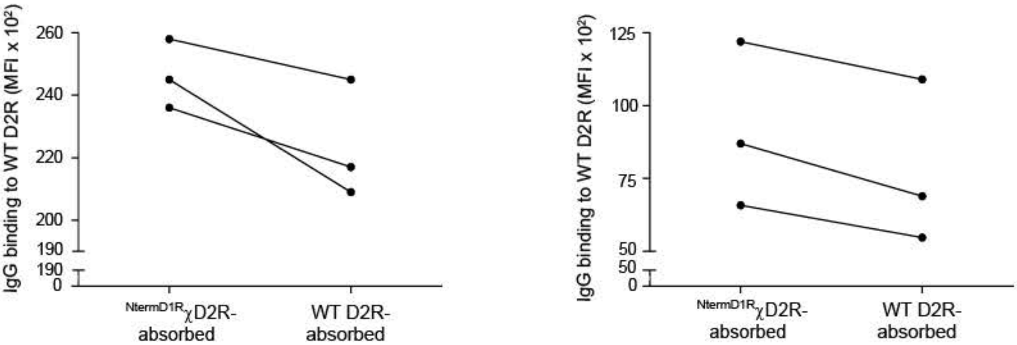

Supplementary Fig. S2

Introduction Figure S3

Commercial extracellular N-terminus-targeting anti-human D2R monoclonal antibody immunolabeled live WT D2R with and without HA tag, suggesting the epitope of lies within amino acids 2-22 and confirming that the HA tag needs to be used to ensure surface recognition of mutants.

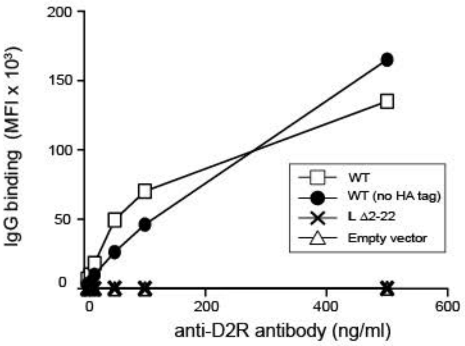

Supplementary Fig. S3

Introduction Figure S4

Point mutations of D2R N-glycosylation sites significantly impair D2R trafficking to cell surface. Green = putative N-glycosylation sites; Red = N to Q point mutation.

a

```
               1              10              20              30
WT             MDPLNLSWYD DDLERQNWSR PFNGSDGKAD RPHYN
N5Q/N17Q/N23Q  MDPLQLSWYD DDLERQQWSR PFQGSDGKAD RPHYN
```

b

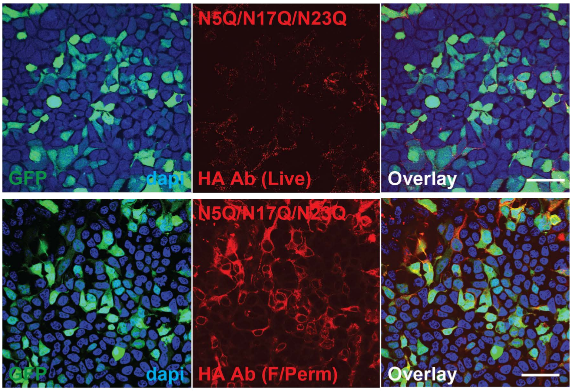

Supplementary Fig. S4

Introduction Figure S5

D2R extracellular N-terminal sequence alignment of different species including human (P14416), mouse (P61168), rat (P61169), chimpanzee (H2R3M5), green monkey (P52702), cow (F1N4I3), pig (F1SM69), European ferret (Q6TLI9), dog (Q9GK99), chicken (A9YZQ5), African clawed frog (P24628), and Japanese pufferfish (P53453). N-glycosylation sites are shown in green highlights.

N-Glycosylation site
Mammalian Conserved sequences

D2R extracellular N-terminal domain

Homo Sapiens **Human**	1	MDPLNLSWYDD-DLERQNWSRPFNGSDGKADRPHYNYY	37
Mus musculus **Mouse**	1	MDPLNLSWYDD-DLERQNWSRPFNGSEGKPDRPHYNYY	37
Rattus norvegicus **Rat**	1	MDPLNLSWYDD-DLERQNWSRPFNGSEGKADRPHYNYY	37
Pan troglodytes **Chimpanzee**	1	MDPLNLSWYDD-DLERQNWSRPFNGSDGKADRPHYNYY	37
Chlorocebus aethiops **Green monkey**	1	MDPLNLSWYDD-DLERQNWSRPFNGSDGKADRPHYNYY	37
Bos taurus **Cow**	1	MDPLNLSWYDD-DPESRNWSRPFNGSEGKADRPPYNYY	37
Sus scrofa **Pig**	1	MDPLNLSWYDD-DLESRNWSRPFNGSEGKADRPHYNYY	37
Mustela putorius furo **European ferret**	1	MDPLNLSWYDD-DPESRNWSRPFNGSEGKVGKPHYNYY	37
Canis lupus familiaris **Dog**	1	MDPLNLSWYDD-DLESQNWSRPFNGSEGKPGKPHYNYY	37
Gallus gallus **Chicken**	1	MDPLNLSWYNS-G--DRNWSKPLNESSA-DQKPQYNYY	34
Xenopus laevis **African clawed frog**	1	MDPQNLSMYND-DINNGT------NGTAVDQKPHYNYY	31
Takifugu rubripes **Japanese pufferfish**	1	MDVFTQYAYNDSIFDNGTWSA---NETTKDETHPYNYY	35

Supplementary Fig. S5

	20 29
**Human D2R**	NWS**RPFN**GS**D**GK**A**D
**unnamed protein product [Penicillium roqueforti FM164]**	FLG**RPFNG**QDGV**A**T
	55 64

Supplementary Fig. S6
